# Processing Facial Expressions That Conflict With Their Meanings to an Observer: An Event Related Potential Study

**DOI:** 10.3389/fpsyg.2020.01273

**Published:** 2020-06-17

**Authors:** Qiwei Yang, Yuping Zhang, Jianfeng Wang, Yan Wu

**Affiliations:** Sichuan Research Center of Applied Psychology, Chengdu Medical College, Chengdu, China

**Keywords:** facial expression, outcome evaluation, event-related potential, N1, P3

## Abstract

As social signals, identical facial expressions can be perceived differently, even oppositely, depending on the circumstances. Fast and accurate understanding of the information conveyed by others’ facial expressions is crucial for successful social interaction. In the current study, we used electroencephalographic analysis of several event-related potentials (ERPs) to investigate how the brain processes the facial expressions of others when they indicate different self-outcomes. In half of the trial blocks, a happy face indicated “Win” and an angry face indicated “Lose.” In the other half of the blocks, the rule was reversed. The results showed that the N170 could distinguish expression valence and the N300 could distinguish outcome valence. The valence of the expression (happy or angry) and the valence of the outcome (Win or Loss) interacted with each other in the early, automatic perceptual processing stage (N1) as well as in the later, cognitive evaluation stage (P300). Standardized Low-Resolution Electromagnetic Tomography (sLORETA) results indicated that the N1 modulation only occurred for happy faces, which may relate to automatic emotion regulation, while the interaction on P300 was significant only for angry faces, which might be associated with the regulation of negative emotions.

## Introduction

The facial expressions of others convey information that is important for social communication. The processing of facial expression has been found to be strongly modulated by situational context such as the emotional valence of background images (Carroll and Russell, 1996), the meaning conveyed by stories accompanying facial expressions (Righart and de Gelder, 2006), and the race (Herzmann et al., 2013), attractiveness (Liang et al., 2010), and trustworthiness (Ruz et al., 2013) of people whose faces are being viewed.

As a social signal, the same facial expression can be perceived differently depending on these influencing factors. This phenomenon could be assumed in two ways. First, how much does the expression on a perceived face influence the attentional resources that it can attract? For example, when someone is in a singing competition, even though the audience includes hundreds of faces, the judges’ faces are the center of one’s attention because their facial expressions are valid predictors of one’s score. Second, what is the relationship between the valence of the expression itself and the valence of the meaning it conveys? A happy face of a partner indicates one’s team is winning. In this case, both the valence of the facial expression and the valence of its outcome to him/her are positive. However, when the face of one’s opponent is frustrated, its outcome is also positive for him/her, despite the negative valence of the expression itself. In order to integrate a facial expression in a particular outcome, we must check whether its outcome valence and its specific emotional valence are contextually appropriate. According to previous studies, the processing and decoding of facial expressions of emotion involves a double check of valence and specific emotional information for the perceiver (Aguado et al., 2013, 2019). However, how the valence of a perceived emotion and the valence of the self-outcome it conveys are processed in the brain has not yet been explored.

The main goal of the present study is to investigate how the brain processes the facial expressions of others when they indicate different self-outcomes with electrophysiological recording. In the current study, different valences of facial expressions (happy and angry) were used to indicate the outcomes in a monetary gambling game. A participant was presented with two rectangles on the screen, one associated with a positive outcome (Win) and the other associated with a negative outcome (Loss). After they selected a rectangle, a picture of facial expression would appear to reveal the outcome. In half of the trial blocks, a happy face indicated “Win” and an angry face indicated “Lose.” In the other half of the blocks, the rule was reversed. Four conditions were created: (matched conditions) valence of the face and valence of the outcome were both positive or both negative (Happy face indicated Win; Angry face indicated Loss); (mismatched conditions) valences were opposite (Happy face indicated Loss; Angry face indicated Win). Before each block, participants were instructed as to which pairing would be used. The even-related potentials (ERPs) obtained during these different conditions were then compared.

Based on the abundant evidence from affective priming studies (Fazio et al., 1986; Moors and De Houwer, 2001; Klauer and Musch, 2003), we assume that the valence of perceived emotion checking is automatic, taking place at early processing stages. According to previous studies about outcome evaluation (Wu and Zhou, 2009; Yang et al., 2018), we assume that the valence of outcome checking is intentional, taking place at later processing stages. A general prediction that directly follows this account is that the valence matching between facial expressions (happy/angry) and outcome (win/lose) should have differential effects on the processing of positive and negative expressions.

Numerous ERP studies have investigated the time course of facial expression processing (Werheid et al., 2005; Trautmann et al., 2009; Vlamings et al., 2009; Lassalle and Itier, 2013; Zhang et al., 2013; Recio et al., 2014; Yuan et al., 2014). Several ERP components have been consistently observed. N100 (the fronto-central distributed negative component) and P100 (the parietal positive component) reflect very fast, automatic early perceptual processing of faces. N170 (the negative parietal-occipital component) is specifically elicited by faces and is sensitive to affective valence. The fronto-central vertex-positive potential (VPP), N300, and P300 are components that reflect the differentiation and evaluation of various facial expressions (Luo et al., 2010). The present study hypothesized that among the ERP components usually elicited by facial expressions, P300 would be selectively modulated by the outcome (Win or Lose). P300 is often modulated by the emotional or arousing content of stimuli. Studies have shown that compared with neutral stimuli, emotional stimuli enhanced the P300 component, and this modulation was stronger for highly arousing stimuli (Carretie et al., 1997; Cramer, 1998). Additionally, this component is thought to reflect evaluative processing, such that its amplitude increases when more cognitive resources are allocated (Friedman et al., 2001; Wu and Zhou, 2009; Asaumi et al., 2014; Roca et al., 2015). We assume that in the conditions for which the two valences are inconsistent, increased cognitive resources would be demanded, which would contribute to a larger P300 than when the two valences were consistent. We also hypothesize that ERP discrimination of the expressions would be earlier than that of the outcomes because the participants need to recognize the expression before they can know the outcome. Further, a recent ERP study found that early perceptual components such as P100 were also sensitive to social-emotional regulation, supporting the flexibility and modifiability of early ERP components (Beckes et al., 2013). Therefore, we hypothesize that an interaction between the two valences also occur between the early components such as N1 and P1.

## Materials and Methods

### Participants

Twenty right-handed participants with no history of neurological disorders, brain injury, or developmental disabilities participated in the experiment. All had normal or corrected-to-normal vision. The study was approved by the Medical Ethical Committee of Shenzhen University. All participants provided their written informed consent. Data from two participants were excluded because the percentage of bad electroencephalographic (EEG) epochs was too high (35%). Thus, 18 participants were included in the final analysis (10 men; age: 24.95 ± 0.65 years).

### Stimuli

The stimuli used comprised 120 photos of faces from the native Chinese Facial Affective Picture System (CFAPS), including 60 happy faces and 60 angry faces. The recognition consistency was 86.64 ± 8.38% for happy expressions and 83.77 ± 6.56% for angry expressions. The intensity of happy and angry expressions was 6.43 ± 0.86 and 6.78 ± 0.69, respectively. No significant differences of recognition accuracy or intensity were found between the two categories of faces (*p* > 0.5). Faces of men and women were represented equally. Happy and angry faces were identical to each other in size, background, contrast grade, brightness, and other physical properties. All faces were gray-scale and were presented on a black background (3.0° × 3.5° visual angle).

### Experimental Procedures

**Figure 1:**
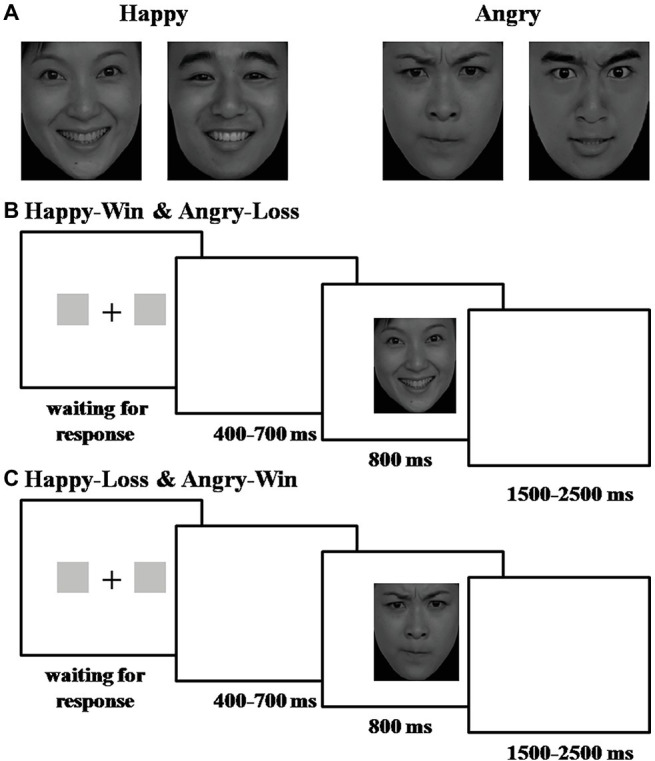
Experimental procedure and stimuli. **(A)** Examples of the photos used: (left) happy male/female faces; (right) angry male/female faces. The faces were selected from the revised version of the Chinese Facial Affective Picture System (CFAPS). **(B)** Experimental procedure for Happy-Win and Angry-Loss blocks. **(C)** Experimental procedure for Happy-Loss and Angry-Win blocks.

Stimulus presentation and behavioral data acquisition were performed using E-Prime software (Version 1.0, Psychology Software Tools, Inc.). During the task, participants sat comfortably in an electrically-shielded room approximately 100 cm from a 15-inch color computer monitor. Each trial began with the individual presentation of two gray rectangles (2.3° × 3.2° of visual angle), which indicated two alternative options on the left and right sides of a fixation point. The participant was informed that one rectangle corresponded to a “Win” and the other to a “Loss.” The participant was asked to gamble by pressing the “F” or “J” key on a keyboard with their index fingers to choose one rectangle. The rectangles remained on the screen until the participant chose a side. Next, a blank interval lasting 400–700 ms (randomly) was presented, followed by the presentation of a face at the chosen location that represented the outcome. The photo remained on the monitor for 800 ms. The inter-trial interval varied from 1,500 to 2,500 ms (see Figure 1).

Trials were presented in four blocks of 120 trials (total 480 trials). Before two of the four blocks, participants were informed that a happy face indicated a “Win” and an angry face indicated a “Loss.” In the other two blocks, they were told the reverse. Block order was counterbalanced across participants. Participants were informed that each trial was worth 10 renminbi (RMB) (i.e., they could win or lose 10 RMB on each trial).

We used a 2 × 2 within subject experimental design. The first factor was the valence of the facial expressions: Happy or Angry. The second factor was the valence of the outcome indicated by the face: Win or Loss. There were four conditions: Happy-Win, Happy-Loss, Angry-Win, and Angry-Loss.

Before the experiment, the task, its rules, and meaning of the faces were explained to the participants. Additionally, they were told that the higher the points they earned, the more bonus money they would receive at the end of the experiment. However, after the task, they were briefed that their total gains and losses were balanced.

### Electroencephalography Acquisition and Analysis

Electroencephalographic data were recorded from a 64-electrode scalp cap using the 10–20 system (Brain Products, Munich, Germany) with the reference on the left and right mastoids. A vertical electrooculogram (EOG) was recorded with electrodes placed above and below the left eye. EEG and EOG data were amplified, band-pass filtered (0.01–100 Hz), and sampled at 500 Hz. All electrode impedances were maintained below 5 kΩ.

EEG data were pre-processed and analyzed using MATLAB R2011b (Math Works, US) and EEGLAB toolbox (Delorme and Makeig, 2004). EEG data at each electrode were down-sampled to 250 Hz, re-referenced to the grand average, and band-pass filtered (0.01–30 Hz). EEG data from 200 ms before until 800 ms after the onset of the facial stimuli were extracted. In order to discard data that was contaminated by EOG artifacts, the data were decomposed by extended infomax ICA using binica, as implemented in EEGLAB (Jung et al., 2001). Epochs with amplitude values exceeding ± 50 μV at any electrode were excluded from the average.

### Data Measurement and Analysis

We mainly analyzed the ERP elicited by happy and angry faces. The averaged epoch was 1,000 ms, including a 200 ms pre-stimulus baseline. In this study, the amplitudes of N1, P1, VPP, N170, N300, and P300 components were measured and analyzed. Based on the topographical distribution of the grand-averaged ERP activity and previous studies (Righart and de Gelder, 2006; Williams et al., 2006; Luo et al., 2010), different sets of electrodes for each component were chosen. Fz, F3, F4, FCz, FC3, and FC4 electrode sites were selected for the analysis of N1 (90–140 ms) and VPP (140–220 ms); Pz, P3, P4, POz, PO3, and PO4 were selected for the analysis of P1 component (100–160 ms); N170 component (140–200 ms) was analyzed at the P7, P8, PO7, and PO8 electrode sites; N300 component (250–400 ms) was analyzed at the T7, T8, FT7, and FT8 electrode sites; and 10 electrode sites (Cz, C3, C4, CPz, CP3, CP4, Pz, P3, P4, and POz) were selected for the statistical analysis of P300 component (300–500 ms). A three-way repeated measure analysis of variance (ANOVA) on the amplitude of each component was conducted with Face pictures (two levels: Happy, Angry), Outcome (two levels: Win, Loss), and Electrode site as within-subject factors. Degrees of freedom for F-ratios were corrected according to the Greenhouse-Geisser method. Statistical differences were considered significant at *p* < 0.05; *posthoc* comparisons were Bonferroni-corrected at *p* < 0.05.

### sLORETA Analysis

We used Standardized Low-Resolution Electromagnetic Tomography (sLORETA) to determine the sources of the differences that we found in the N100 and P300 components. sLORETA is a functional imaging method based on certain EEG and neuroanatomical constraints (Pascual-Marqui et al., 1994). It computes images of electrical activity from the EEG data in a realistic head model using the MNI152 template and estimates the three-dimensional distribution of the current density within 6,239 voxels at a spatial resolution of 5 mm. This method has been established as useful for determining deep structures such as the ACC and others within the temporal lobe (Pizzagalli et al., 2004; Zumsteg et al., 2006).

For the current dataset, in order to localize the brain structures responsible for the effects we observed on N100 and P300, a *t*-test was performed for the current densities on different conditions for N100 and P300 in their respective time windows (N100: 90–140 ms; P300: 300–500 ms), employing a LOT-F-ratio statistics for paired groups (Happy-Loss > Happy-Win for N100 and Angry-Win > Angry-Loss for P300, separately), with 5,000 bootstrapping and a level of significance of *p* < 0.05.

## Results

### N100

**Figure 2:**
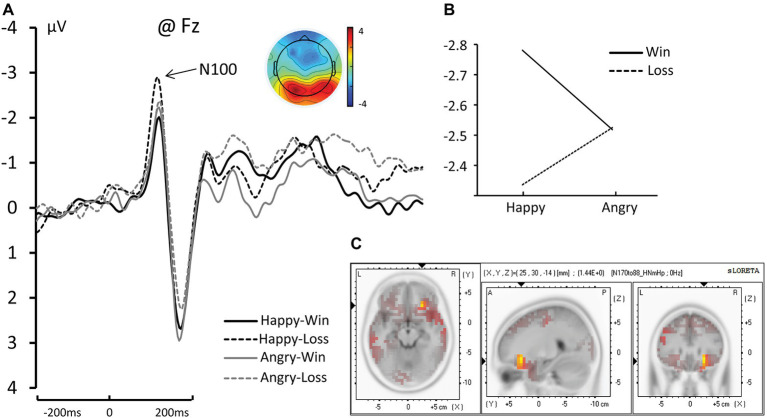
**(A)** The grand average and scalp topography of N100 component at Fz site for four conditions [Happy-Win (black lines); Happy-Loss (black dotted line); Angry-Win (gray line); Angry-Loss (gray dotted line)]. **(B)** The interaction of Face × Outcome on N100. **(C)** Standardized Low-Resolution Electromagnetic Tomography (sLORETA) results of “Happy-Loss” > “Happy-Win” in time windows of N1.

The Face (Happy vs. Angry) × Outcome (Win vs. Loss) interaction was significant for N100 amplitude [*F*_(1, 17)_ = 5.433, *η_p_*^2^ = 0.242, and *p* = 0.032]. The pairwise comparisons revealed that when the facial expression was Happy, Losses elicited significantly greater negative amplitude than Wins (−2.781 μV for Happy-Loss and −2.336 μV for Happy-Win, *p* = 0.005). The difference between Win and Loss was not significant for angry faces (−2.518 μV for Angry-Loss and −2.525 μV for Angry-Win, *p* = 0.0968; see Figure 2).

### N170

**Figure 3:**
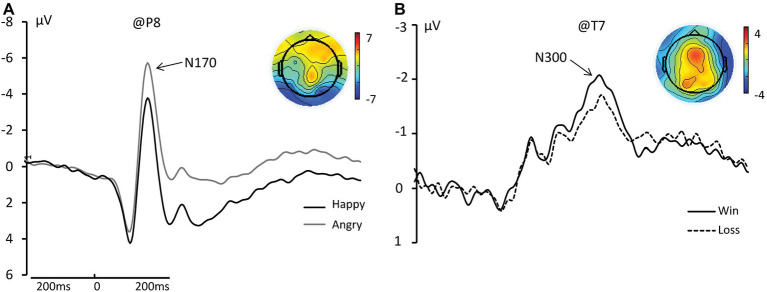
**(A)** The grand average and scalp topography of N170 component at P8 site for two conditions [Happy (black lines); Angry (gray line)]. **(B)** The grand average and scalp topography of N300 component at T7 site for two conditions [Win (black lines); Loss (black dotted line)].

We found a significant main effect of face on N170 amplitude such that angry faces elicited significant larger amplitudes than happy faces. [Happy: −5.922 μV; Angry: −6.258 μV; *F*_(1, 17)_ = 7.457, *η_p_*^2^ = 0.305, and *p* = 0.014]. We did not find a main effect of outcome or an interaction between Face and Outcome (see Figure 3A).

### N300

We found a significant main effect of Outcome for N300 amplitude such that Wins elicited significantly greater negative amplitudes than Losses [Wins: −4.177 μV; Losses: −3.710 μV; *F*_(1, 17)_ = 10.848, *η_p_*^2^ = 0.390, and *p* = 0.004; see Figure 3B].

### P300

**Figure 4:**
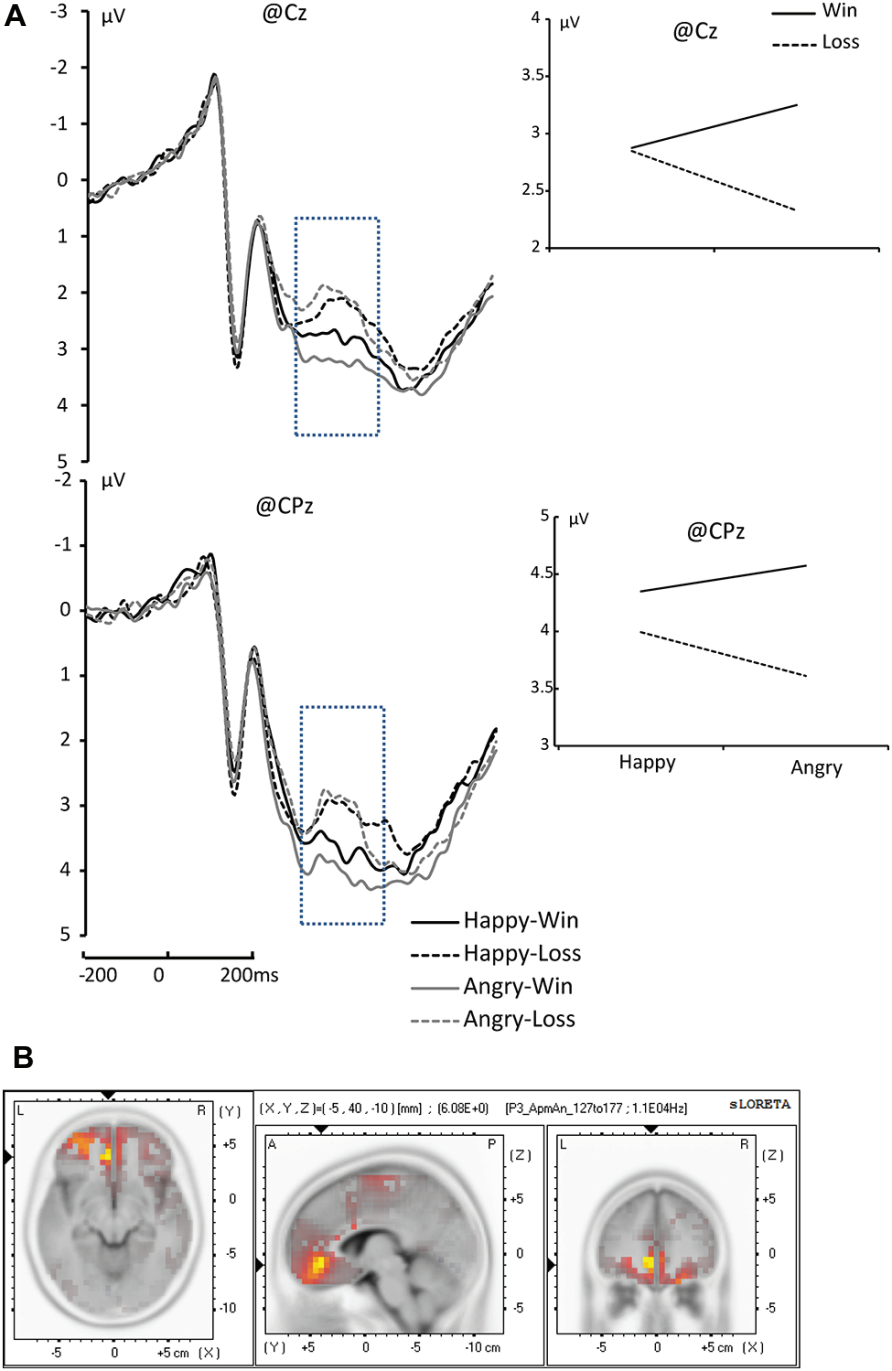
**(A)** The grand average of P300 component at Cz and CPz, [Happy-Win (black lines); Happy-Lose (black dotted line); Angry-Win (gray line); Angry-Lose (gray dotted line)]. **(B)** sLORETA results of “Angry-Win” > “Angry-Lose” in time windows of P3 (marked with blue dotted rectangles in the waveforms).

We found a significant main effect of Outcome on P300 amplitude. Wins elicited significant larger amplitudes than Losses [Wins: 4.257 μV; Losses: 3.950 μV; *F*_(1, 17)_ = 11.004, *η_p_*^2^ = 0.393, and *p* = 0.004]. We also found a significant main effect of Electrode [*F*_(9, 153)_ = 6.618, *p* < 0.001]. Specifically, FCz, FC3, FC4, Cz, C3, C4, and Pz electrodes elicited larger amplitudes than the others (*p* < 0.05). Additionally, we found that the three-way interaction of Face × Outcome × Channel was significant [*F*_(9, 153)_ = 3.283, *η_p_*^2^ = 0.162, and *p* = 0.016]. Pairwise comparison revealed that significantly larger amplitudes occurred for angry faces on Wins than on Losses at Cz, C3, C4, CPz, and CP3 (*p* = 0.001, *p* = 0.006, *p* = 0.031, *p* = 0.013, and *p* = 0.004, respectively; see Figure 4A).

We did not find any significant main effects or interactions for other ERP components.

### Standardized Low-Resolution Electromagnetic Tomography

The analyses revealed a difference in the inferior frontal gyrus and middle frontal gyrus (BA47/BA11) between Happy-Loss and Happy-Win conditions within the N1 time window such that the Happy-Loss condition resulted in significantly higher current density than the Happy-Win condition [Montreal Neurological Institute (MNI) coordinates = (25, 30, −14), *p* = 0.017; see Figure 2C]. The Angry-Win resulted in significantly higher activation in the anterior cingulate cortex (BA32) and orbitofrontal region (BA11) than did the Angry-Lose condition within the P300 time window (MNI = −5, 40, −10, *p* = 0.018; Figure 4B).

## Discussion

This study explored how the brain processes facial expressions that indicate monetary outcome for oneself. Our results show that the brain first distinguishes the valence of the expression (happy from angry in our case), as reflected in a significant main effect of the facial expression in the N170 component. The brain also distinguishes the outcome, as reflected in the observed significant main effect of outcome on N300. The processing of self-outcome interacted with the processing of facial expression in the early automatic stage and also in the later evaluative stage, as reflected by the observed significant interactions that affected N1 and P300 amplitude.

### The Interaction in the Early Automatic Stage of Processing: N100

In the frontal N1, we found a significant interaction of Face × Outcome in which the difference in amplitude between Win and Loss was significant for a happy face, but not for an angry face. Specifically, the Happy-Loss condition elicited a significantly larger negativity than the Happy-Win condition. This result indicates that the outcome for the observer began to interact with the perception of the facial expression at a very early, automatic stage. The enhancement during the Happy-Lose condition could be related to an initial amplification of relevant processing generated by top-down factors (Hillyard and Picton, 1987; Ruz et al., 2013). In the mismatched blocks, facial expressions were not tied to their natural meanings. Therefore, the recognition of the outcome from the facial expression was likely associated with higher processing demands, which requires the allocation of more cognitive resources (Williams et al., 2003). The sLORETA results revealed a significant difference between Happy-Loss and Happy-Win which was related to higher activation in the orbitofrontal cortex (BA11) in the Happy-Lose condition than in the Happy-Win condition. This brain region has been found to be specifically activated in automatic emotion regulation (ER) (Ochsner and Gross, 2005; Phillips et al., 2008; Etkin et al., 2011; Hallam et al., 2015). The automatic ER seems to underlie this processing. ER refers to the processes involved in the initiation, maintenance, and modification of the occurrence, intensity, and duration of feeling states (Gross and Levenson, 1993; Eisenberg et al., 2000). Automatic ER specifically means the ER with features of automaticity (i.e., immediacy, efficiency, and redundancy of conscious intent (Gollwitzer and Sheeran, 2006). The brain regions that support automatic ER include medial frontal areas such as the medial orbitofrontal cortex (mOFC). Based on the literature, the mOFC is a heteromodal association area that unites information from the sensory modalities, representations of past experiences, and the processing of contextually-relevant information (Hallam et al., 2015). It is thus suitable for handling the expressions of others that convey information with changing valences, which requires the integration of multiple types of information, such as those from sensory input, experience, and social contexts.

Why the Win/Loss difference was only significant for happy faces was not immediately clear. It might be related to the lower priority that happy faces have in social interactions. Studies suggest that angry expressions are initially prioritized by our cognitive system because we benefit from early detection of potential threats in the environment (Fox et al., 2000; Avero and Calvo, 2006). However, unlike detection tasks, happy expressions show clear advantages in recognition tasks. Happy faces were found to be recognized faster and more accurately (Leppanen and Hietanen, 2004). The same study also found that a smiling mouth became visually salient very early (~95 ms), which corresponds temporally with the N100 (Calvo et al., 2014). Another study showed that among all expressions, only recognition of happy expressions was unaffected by the intensity of the expressions—even low intensity happy faces were recognized with nearly 100% accuracy (Hess et al., 1997). In situations in which happy faces indicate a negative outcome, they would likely be quickly recognized and then modulated through automatic ER. Angry faces might not yet be recognized during this time window.

### The Discrimination of the Two Valences: N170 and N300

N170 is a negative-going component detected at the occipito-temporal electrode sites that peaks around 170 ms post-stimulus. The component clearly distinguishes faces from non-face visual stimuli. However, evidence regarding whether N170 is responsive to emotional expression is conflicted; while some studies found that N170 did not discriminate emotional expressions (Luo et al., 2010; Nakajima et al., 2012), others found that it did (Batty and Taylor, 2003; Miyoshi et al., 2004; Lynn and Salisbury, 2008; Herbert et al., 2013). In particular, N170 amplitude has been reported to differ between happy and angry faces (Krombholz et al., 2007). In line with these latter studies, here we found a main effect of facial expression on N170 in which angry faces elicited significantly larger amplitudes than happy faces.

After decoding the facial expressions, the valence of the outcome could be distinguished *via* the N300 component; we found significantly larger negative amplitude for Wins than for Losses. The N300 largely reflects the dimensionality of the affective valence in higher-level phases of cognitive processing, such as stimulus evaluation and selection (Carretie et al., 2001a,b; Campanella et al., 2002; Luo et al., 2010). In the current study, the participants needed to mentally recognize and label the presented facial expressions, then deduce the monetary outcome. Thus, a main effect of facial expression (N170) before a main effect of outcome (N170) was a reasonable observation.

### The Interaction in the Evaluation Stage: P300

Scientists believe that P300 is involved in a large number of cognitive and affective processes and it is traditionally associated with the allocation of mental resources (Olofsson et al., 2008). When a facial expression contains information that is important to an observer (e.g., monetary gain or loss), it usually draws more attention and requires more cognitive resources to analyze and evaluate. Interestingly, in the current study, we found that during the P300 time window, the positive and negative facial expressions were evaluated differently under different conditions. A three-way interaction of Face × Outcome × Channel was observed. The difference between Angry-Win and Angry-Loss was significant in the central regions. sLORETA results found that regions that were differentially activated between Angry-Win and Angry-Loss were localized in the ACC (BA32) and orbitofrontal region (BA11). These regions have been found to be responsible for the regulation of negative emotions (Levesque et al., 2003; Ochsner et al., 2004, 2012; Phan et al., 2005; Mak et al., 2009). In the current design, in the blocks where the angry face indicated a positive outcome, the participant may need to suppress the negative affect aroused by the naturally negative stimulus and re-identify the face as positive. Thus, recruiting neural circuits related to the regulation of negative affect is unsurprising for this condition.

Other studies have shown that valence can also affect later components, such as, P3 (Olofsson et al., 2008). Interestingly, we did not observe a significant effect of facial expression on the ERPs for which a main effect of expression has often been found (e.g., N300 and P300). We assume this was because the most important information for the participants was not the expressions themselves, but the monetary outcome. Therefore, after recognizing the expressions in the N200 time window, processing of the outcome likely dominated and the effect of the expressions during the N300 and P300 time windows would be weakened.

Actually, ERP studies have produced ambiguous results on the time course of face and valence processing. Some research have found that the P1 and N1 can be modulated by emotional valence (Levesque et al., 2003; Ochsner et al., 2004, 2012; Phan et al., 2005; Mak et al., 2009). Rellecke and colleagues found that automatic enhanced encoding of angry faces were indicated by P1, N170, and EPN in the early processing stages. However, our results only found the main effect of emotional valence in N170 and the valence and outcome interactions with the processing of other’s facial expression in an early automatic stage (Levesque et al., 2003; Ochsner et al., 2004, 2012; Phan et al., 2005; Mak et al., 2009). Let us note that this early P1 modulation by emotion is debated as many studies also failed to report modulations of the P1 by facial expressions of emotion (Levesque et al., 2003; Ochsner et al., 2004, 2012; Phan et al., 2005; Mak et al., 2009). We assumed that the reason is that these components are related to differentiation of certain expressions (Olofsson et al., 2008), which should occur after valence processing according to the dimensional model.

In conclusion, the current investigation explored how facial expression stimuli are processed when they indicate positive or negative outcomes for those observing them. The results suggest that early perceptual processing of facial expression is influenced by the valence of outcomes, as evidenced by an enhanced N100 component when happy faces indicate a financial loss. Subsequently, the valence of the face is decoded by the N170 component and the valence of the outcome is discriminated by the N300 component. At a later cognitive evaluation stage, the face and outcome valences interact again, as evidenced by the differences in the P300 component between financial gains and losses represented by angry faces. This interaction may reflect the regulation of emotional responses that are elicited by negative stimuli when the stimuli indicate positive outcomes.

The sample size (*n* = 18) was a limitation of the current study as it is relatively small for an ERP study. Our findings should therefore be validated using larger sample sizes.

## Data Availability Statement

The datasets generated for this study are available on request to the corresponding author.

## Ethics Statement

The studies involving human participants were reviewed and approved by the Medical Ethical Committee of Shenzhen University. The patients/participants provided their written informed consent to participate in this study.

## Author Contributions

Conceived and designed the experiments: QY and YW. Performed the experiments: YZ and JW. Analyzed the data: JW. Wrote the manuscript: QY and YW.

## Conflict of Interest

The authors declare that the research was conducted in the absence of any commercial or financial relationships that could be construed as a potential conflict of interest.
